# Facial rejuvenation using photodynamic therapy with a novel preparation of ALA and hyaluronic acid in young adults

**DOI:** 10.1007/s00403-020-02038-5

**Published:** 2020-02-14

**Authors:** Alisen Huang, Julie K. Nguyen, Evan Austin, Andrew Mamalis, Marc Cohen, Boris Semkhayev, Derek Ho, Jared Jagdeo

**Affiliations:** Department of Dermatology, State University of New York, Downstate Health Sciences University, 450 Clarkson Ave, Box 46, Brooklyn, NY USA

**Keywords:** Facial rejuvenation, Photodynamic therapy, Hyaluronic acid

## Abstract

Photodynamic therapy (PDT) is a well-established, non-invasive treatment modality that has shown desirable effects such as improvement of fine lines, dyspigmentation, and other signs of photodamage. Many patients seek to decrease, or reverse, effects of sun damage on the skin. Hyaluronic acid (HA), a naturally occurring glycosaminoglycan found in the body, has enormous potential to bind water which allows the skin to retain moisture and maintain elasticity. Topical application of HA has been found to produce anti-wrinkle effects. We conducted a pilot case series evaluating the safety and efficacy of a commercially available 2% 5-aminolevulinic acid (ALA) gel with hyaluronic acid (ALA-HA) and light-emitting diode-red light (LED-RL) for facial rejuvenation as determined by clinical assessments and digital skin analysis of rhytides, pore size, and erythema. All patients noted improvement of their skin and experienced minimal pain during PDT. Evaluation by dermatologists demonstrated significant improvement in overall skin appearance. Digital analysis of photographs revealed improvement of skin spots, wrinkles, pores, texture, UV spots, brown spots, red areas, and porphyrins. Our findings demonstrated safety and efficacy of this novel preparation of photodynamic gel with HA and subsequent activation with LED-RL.

## Introduction

Photodynamic therapy (PDT) is a well-established, non-invasive treatment modality for actinic keratosis [16]. Researchers evaluating the use of PDT for actinic keratosis noted improvement of fine lines, dyspigmentation, and other signs of photodamage, as unexpected but desirable results of the treated areas [9]. This led to clinical investigations of performing PDT for cosmetic skin rejuvenation; however, to date, there exists no standardized treatment guidelines for this indication [25]. PDT utilizes the reaction between a photosensitizer and light to generate reactive oxygen species (ROS) [17]. Prodrugs, such as methyl aminolevulinate (MAL) and 5-aminolevulinic acid (ALA), are converted into protoporphyrin IX (PpIX), a photosensitizer [17]. PpIX is found endogenously but its formation is a rate-limiting step [17]. Topical application of MAL or ALA bypasses this step, increasing intracellular levels of PpIX [17]. Singlet oxygen, a type of ROS, formed by light activation of the photosensitizer is highly reactive, destroying old collagen fibers and inducing the formation of new collagen fibers and subsequently results in skin rejuvenation [15].

Intrinsic aging of the skin is associated with decreased proliferative activity of skin cells including keratinocytes, melanocytes, and fibroblasts [29]. Exposure to light, specifically ultraviolet A (UVA), for extended periods of time, causes extrinsic atrophy of the skin, leading to photodamage and premature aging of the skin [6]. Features of photodamaged skin include wrinkles, discoloration, erythema and sebaceous gland hypertrophy [8]. Previous studies using PDT for treatment of photodamaged skin have found significant histological and clinical improvements including increased collagen, smoother skin texture, and fewer wrinkles [3, 18]. Moreover, hypertrophic scarring has been found to improve with less erythema, reduced volume, and increased flexibility [7].

Many patients seek to decrease, or reverse, effects of sun damage on the skin. According to the Annual Statistics Report by the American Society of Plastic Surgeons, 15.9 million minimally invasive cosmetic procedures were performed in 2018, resulting in a total expenditure of 8.8 billion dollars [23]. According to the American Society for Dermatologic Surgery (ASDS), 3.27 million procedures using laser, light, and energy-based devices were performed in 2017, making those procedures the most popular cosmetic treatment [2]. In addition, a survey done by ASDS in 2018 revealed that treatments to tighten the skin or smooth wrinkles using ultrasound, laser, light, or radiofrequency were the most common procedures that consumers opted to undergo [1].

Herein, we conducted a pilot case series evaluating the safety and efficacy of a commercially available 2% ALA gel with hyaluronic acid (ALA-HA) and light-emitting diode-red light (LED-RL) for facial rejuvenation as determined by clinical assessments and digital skin analysis of rhytides, pore size, and erythema. HA, a naturally occurring glycosaminoglycan found in the body, has enormous potential to bind water which allows the skin to retain moisture and maintain elasticity. Topical application of HA has been found to produce anti-wrinkle effects [19].

## Methods

### Participants

Patients greater than 18 years old and of Fitzpatrick skin types I–VI were able to participate. Patients with Fitzpatrick skin types IV–VI prepped their skin for treatment using a gentle cleanser for 1 week leading up to the first treatment session. All patients provided written informed consent. Exclusion criteria included blood-thinning agents, pregnancy, lactation, open wounds on the face, photosensitive disorders, and photosensitizing medications. IRB approval was not indicated as this is categorized under pilot activity.

### Baseline survey

Prior to treatment, patients rated their degree of facial hyperpigmentation, fine lines and wrinkles, texture and pores using a four-point scale, with 0 being best and 4 being worst.

### Study medication

The photodynamic gel contained hydrogenated lecithin vehicle for delivery of 2% ALA-HA.

### Treatment protocol

Three treatments were administered over 12 weeks (spaced 4 weeks apart). The final follow-up visit was conducted 4 weeks after the last treatment session. All patients washed their face with a gentle cleanser (CeraVe Hydrating Facial Cleanser or LaRoche-Posay Toleriane Hydrating Gentle Cleanser) and then prepped their skin using a 70% alcohol pad and gauze for irritation. A cosmetic photodynamic gel with hyaluronic acid (GlycoALA, GlobalMed Technologies, Glen Ellen, CA) was then applied to the face and left on for 40 min in a dark room. The gel was massaged into the skin by the patient for the first 10 min. After the incubation period in a darkroom, the gel was thoroughly washed off. Patients were exposed to LED-RL (633 nm using the Omnilux Revive 2, GlobalMed Technologies, Glen Ellen, CA) for 20 min. Patients were positioned such that the LED array was approximately two inches from the face (at a standardized distance). Wavelength-specific safety glasses were worn by the patients for the entirety of LED-RL treatment session. Patients were instructed to avoid sunlight and bright lights if possible and to use sunscreen with at least SPF 50 for the next 3 days.

### Clinical assessments and patient-reported outcomes

After LED-RL therapy, patients rated their level of erythema from 0 (none) to 4 (severe), pain from 0 (no pain) to 10 (most severe pain) and commented on any other symptoms during PDT and every day for 7 days after each session. Erythema was also assessed by an observer immediately post-treatment. Both patients and observers used the same scale for erythema. No erythema was scored a 0. Minor or very faint erythema was scored a 1. Mild or blotchy visible erythema that does not cover the whole face was scored a 2. Moderate or a dull red color to the skin was scored a 3. Severe or a bright or dark red color to the skin was scored a 4. Patients were given a diary to track pain from 0 to 10, other discomfort from 0 to 10, and skin redness from 0 to 4 for the next 7 days.

At follow-up visits, patients reassessed their hyperpigmentation, fine lines and wrinkles, texture and pores on a scale of 0–4, with 0 being no improvement and 4 being very noticeable improvement.

### Photographs and digital analysis

Photographs of the frontal facial view were taken at baseline and at the final follow-up. Photographic analysis was completed with the VISIA Complexion Analysis System (Canfield Scientific, Parsippany, NJ). The following skin quality parameters were digitally analyzed: spots, wrinkles, pores, texture, UV spots, brown spots, red areas, and porphyrins. The score for each parameter was generated by the program with higher numbers reflecting greater severity. Scores for each parameter were averaged across all six patients for a baseline score and final follow-up score, which were then compared.

### Photonumeric grading

A modified Global Aesthetic Improvement Scale (mGAIS) was used to grade the photos. Two photos of the same patient, a before and after photo in random order, were presented side by side and could receive a score of − 1, 0, or 1 by the dermatologist graders (AM and JJ). The graders were instructed to compare the second photo to the first photo. A score of − 1 indicated worsened appearance. A score of 0 indicated no change. Finally, a score of + 1 indicated improvement in the appearance. Following grading, the results were decoded chronologically to determine the change in mGAIS from baseline.

### Statistical analysis

The complexion analysis scores after PDT sessions were compared with those at baseline using paired *t* tests. A *p* value of < 0.05 was considered statistically significant.

## Results

### Patient demographics

Six patients (two women, four men, mean age 26.5) completed the full protocol. Two patients had Fitzpatrick skin type II, one patient had Fitzpatrick skin type III, and three patients had Fitzpatrick skin type IV.

### Baseline survey

On the baseline survey, all patients reported visible pores as their most significant concern. The survey revealed an average score of 3.0 for pores, 1.167 for texture, 1.0 for hyperpigmentation and 0.5 for fine lines and wrinkles.

### Clinical assessments and patient-reported outcomes

Average pain on a four-point scale, with 0 being no pain and 4 being the most severe pain, during each of the three sessions, was 0.5 ± 0.5, 1.5 ± 1.3, and 0.83 ± 1.2, respectively. Across all sessions, other reported sensations included tingling, stinging and warmth. Average patient-rated erythema on a four-point scale, with 0 being none and 4 being extensive facial erythema, was 1.17 ± 0.4, 1.5 ± 0.5, and 1.5 ± 0.5 for each of the sessions, respectively. Average clinician-rate erythema on a 4-point scale was 1.17 ± 0.9, 2 ± 0.6, and 1.3 ± 0.5 for each of the sessions, respectively. In the 7 days after LED-RL therapy, pain ranged from 0 to 4 overall.

Patients reported mild improvement from baseline with skin texture (1.5 out of 4) followed by pigmentation (1.17 out of 4) and pores (1.17 out of 4). Overall, no improvement or worsening of fine lines was noted. Half of the patients would recommend this treatment to someone else. Pain, other discomfort and redness were low for the 7 days following treatment for each session. Across all three sessions, the pain level was rated at an average of 0.13, ranging from 0 to 2, other discomfort averaged 0.66, ranging from 0 to 6, and redness averaged 0.56, ranging from 0 to 2. Three out of six patients (50%) reported dryness in the 7 days post-treatment, making it the most commonly reported side effect. Other comments included tingling, itching, soreness, and peeling.

### Digital analysis

**Fig. 1:**
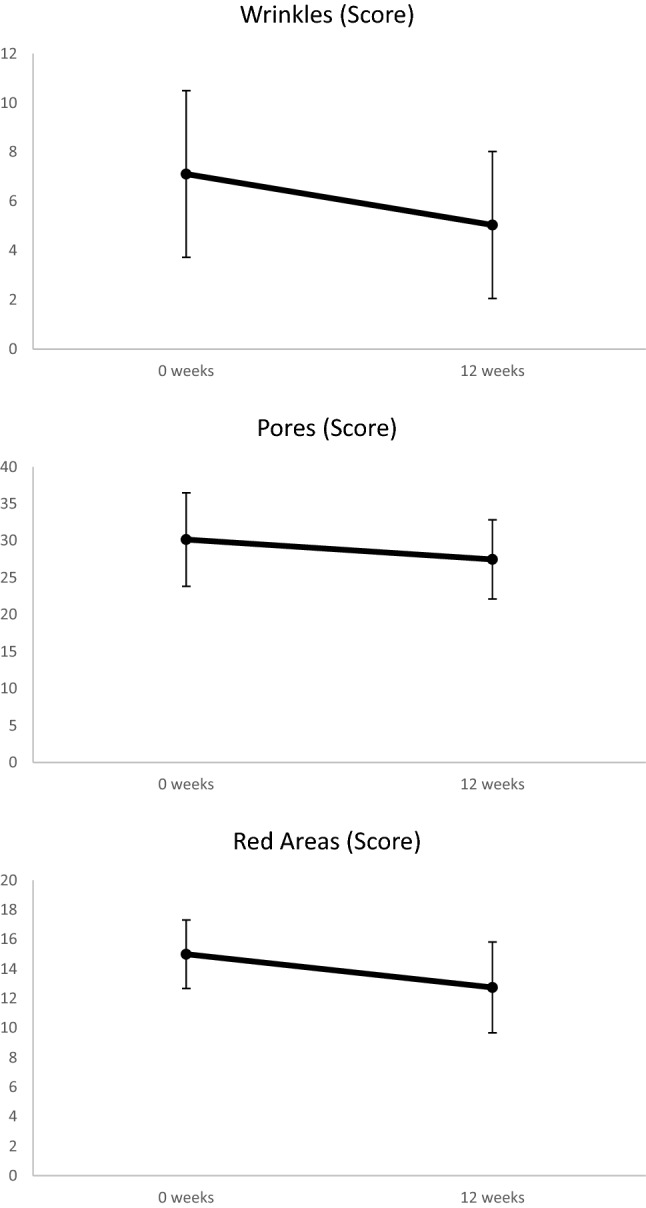
Averaged digital skin analysis showing positive trends (i.e., decreased severity) for wrinkles (**a**), pores (**b**), and red areas (**c**)

**Fig. 2:**
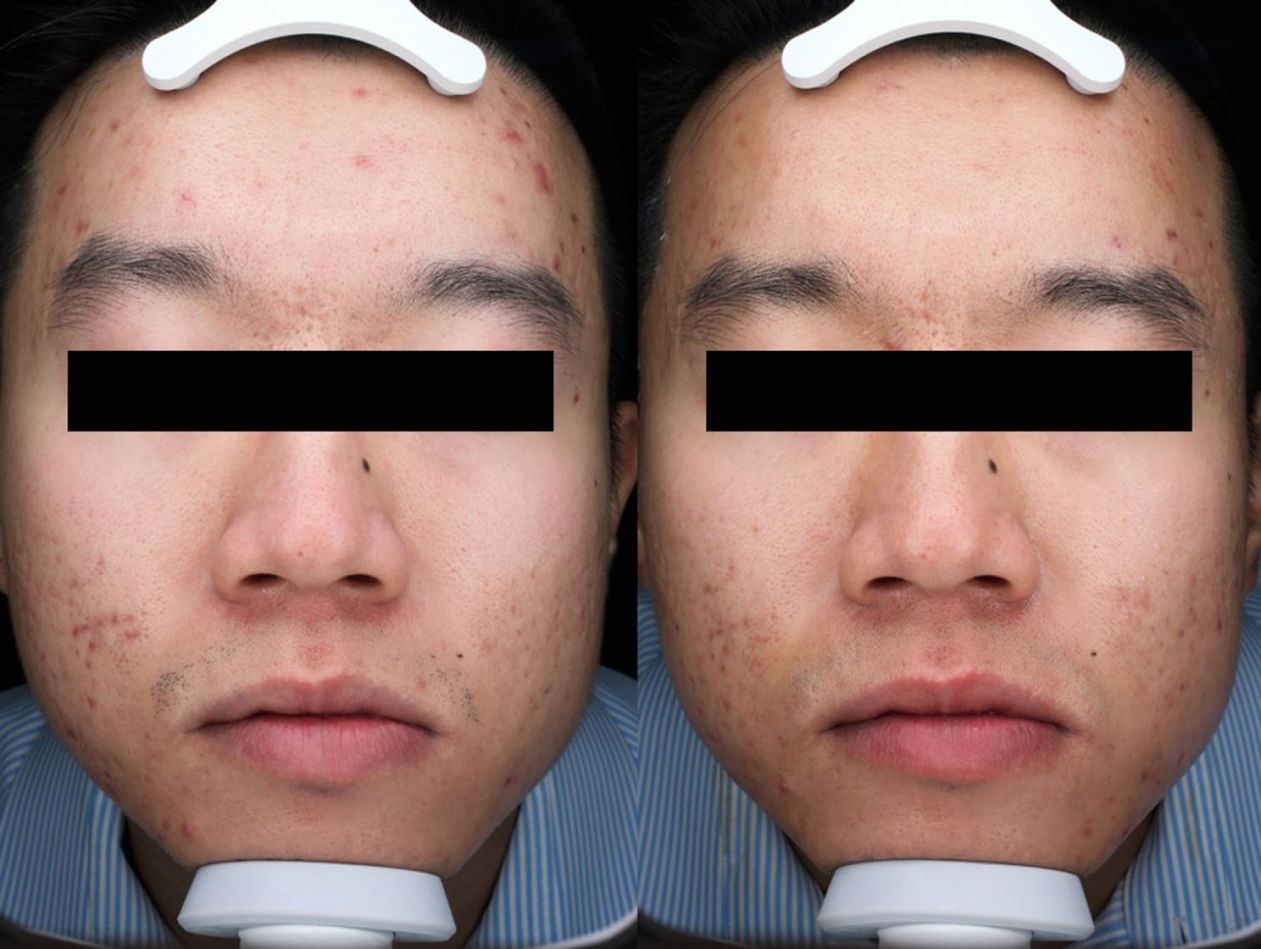
Standard photography of a patient before photodynamic therapy (**a**) with improvement at 4 weeks after last treatment (**b**)

**Fig. 3:**
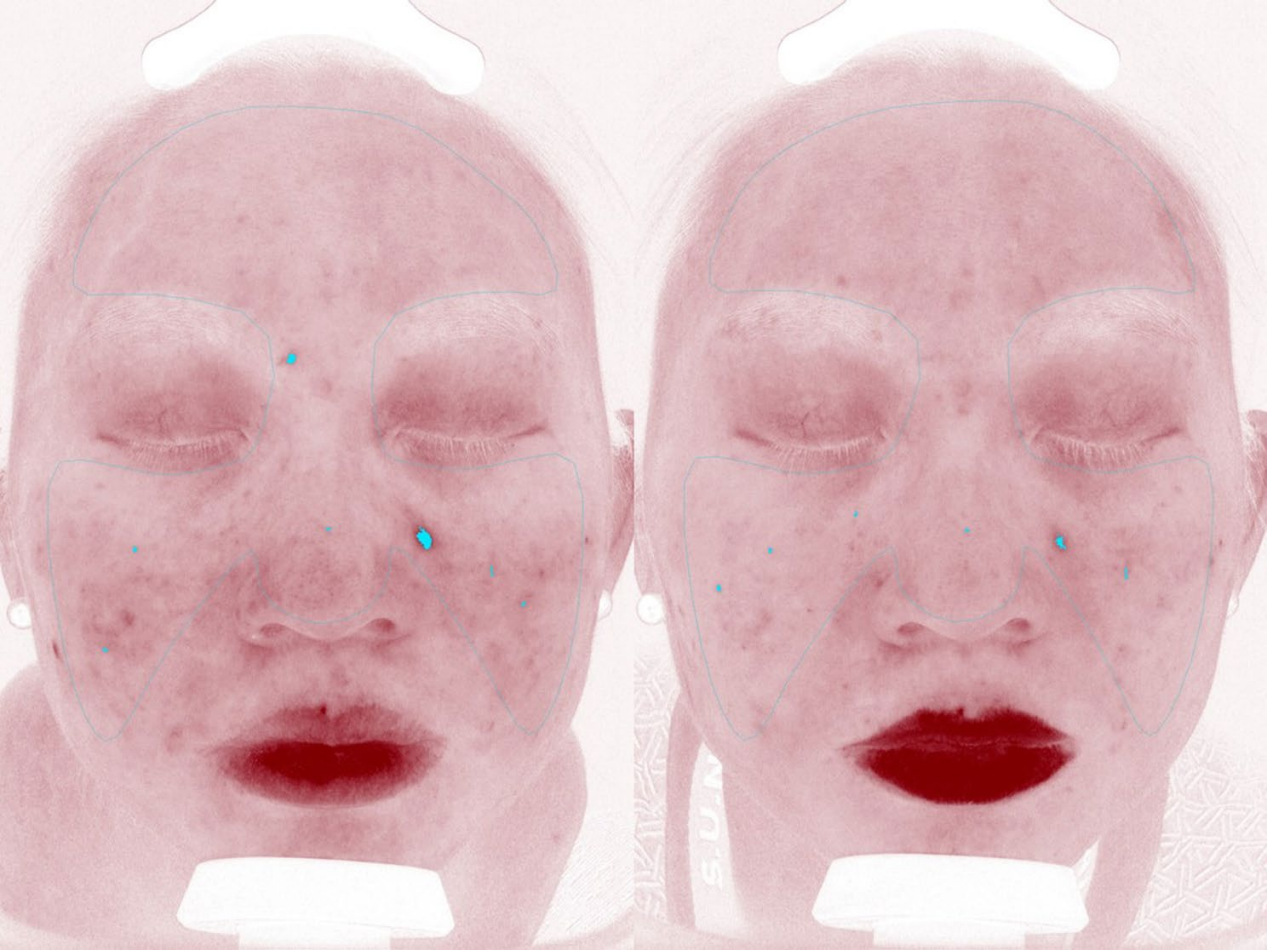
Photography with Red/Brown/X processing to show red areas of a patient before photodynamic therapy (**a**) and at 4 weeks after last treatment with improvement (**b**)

Photographic and digital skin analysis revealed positive trends (i.e., decrease in severity), that were not statistically significant changes, of all analyzed parameters after treatment compared to baseline (Fig. 1). Improvements in wrinkles, pores, and redness appearance were the greatest and approached statistical significance (p = 0.068, p = 0.087, and p = 0.061, respectively; Figs. 1, 2, 3).

### Photonumeric grading

Standard photos at baseline and final follow-up were presented to two dermatologists; Fig. 2 shows an example in chronological order. Four out of six patients (67%) were rated to have improved appearance by dermatologist’s mGAIS. One patient was rated to have worsened appearance, and one patient was rated improved by one physician and worsened by the second physician.

## Discussion

Many individuals seek treatment of photodamage to their facial skin. Patients undergoing PDT for actinic keratosis were noted to have desirable cosmetic outcomes. Our findings demonstrated safety and efficacy of this novel preparation of photodynamic gel with HA and subsequent activation with LED-RL.

Based upon patient-reported outcomes, all patients noted improvement of their skin and 50% of participants would recommend the procedure to someone else after three PDT sessions. Interestingly, the amount of pain reported by the patients was not as high as other studies with PDT, making glycoALA-PDT a comfortable procedure. The main limiting adverse event for patients undergoing PDT is pain, which often limits patient interest and compliance with additional future treatments [26]. The pain experienced during PDT is unpredictable but appears to be associated more with ALA than MAL [4]. PDT pain may be related to rapid activation of PpIX [5]. One study found that ROS activates TRPA1 and TRPV1, cation channels associated with body temperature regulation. These channels are located in nociceptive nerve endings and may be responsible for pain experienced during PDT [5]. Pain may also be related to the amount of photodamage present. The level of pain may be decreased in our cases compared to other studies due to less photodamage in our relatively younger group. In addition, the diluted active ingredient, 2% ALA, as opposed to the 20% ALA typically used to treat actinic keratosis, may play a role in the decreased pain experienced by the participants. Post-PDT session diaries also revealed minimal to moderate erythema and minimal discomfort.

According to blinded reviewers, clinically visible improvement was apparent with most patients from baseline compared with 3-month photos. We believe that hyaluronic acid delivery via PDT allows for a non-invasive therapy that is more affordable and more accessible to the general population than currently available methods such as lasers or injectables.

The digital analysis of photographs by our imaging system demonstrated improvement of skin spots, wrinkles, pores, texture, UV spots, brown spots, red areas, and porphyrins. Wrinkles, pores, and redness parameters approached significance, showing a positive trend. Although not statistically significant, our results were clinically significant and relevant as patients and dermatologists noticed an improvement in overall skin appearance. Patients also reported improvement of skin texture, pigmentation, and pores after treatment.

Our findings are consistent with previous reports. Lowe et al. used 5% ALA for 30 min and subsequent LED-RL for one session [14]. There was minimal phototoxic response with a reduction of fine lines in 67% of patients and increased skin softness in all patients [14]. Another study using 5% ALA for 2 h and then LED-RL exposure found improvement of photoaged lesions, stratum corneum hydration, elasticity, transepidermal water loss, and melanin index. These improvements were more obvious than an intense pulsed laser-only group [28].

Optimizing photodynamic gel and treatment protocol with LED-RL irradiation may result in a new method for desirable antiaging effects. One study using PDT and 0.5% 5-ALA liposomal spray demonstrated an improvement of periorbital wrinkles in Asians [22]. A major drawback of PDT is pain reported by patients during the procedure. Based on our study, patients experienced minimal pain. Another study found ALA-PDT with red light to be better than red light alone for skin rejuvenation [12]. A review of photodynamic photorejuvenation concluded that photorejuvenation PDT is safe, efficient and an integral part of the energy-based technologies and laser rejuvenation techniques; however, optimization of protocols is still needed [13].

MAL-PDT has been effective in decreasing the extent of keratinocyte atypia associated with new dermal collagen deposition [24, 27]. It has also been found to decrease tumor protein p53 and elevate levels of procollagen-I, matrix metalloproteinase (MMP)-1 and tenascin-C [18, 24]. Park and colleagues collected skin biopsies before and after two sessions of ALA-PDT [18]. They found decreased epidermal thickness and decreased inflammatory infiltrate. TGF-β and its receptor, which are involved in collagen synthesis, were increased, while MMP-1, 3, and 12, elastin degraders, were decreased [18]. PDT is associated with improvement of fine wrinkles, texture, and pigmentation [10, 11, 20, 21, 25, 27].

This pilot case series was limited by the small sample size and emphasizes the need for larger studies due to the positive trends of data. Our participants were younger than the typical age included in photorejuvenation studies and the range of skin types was limited. We made efforts to decrease bias by utilizing randomization of grading and digital analysis. A study with larger sample size, longer sessions, additional sessions, or longer follow-up period may be necessary to detect statistically significant differences in skin quality parameters. While PDT is often used in older adults for the treatment of actinic keratosis, it is unclear whether PDT at a younger age has benefits in preventing actinic keratosis formation. PDT treatment with ALA-HA in younger patients may provide a non-invasive alternative for “prejuvenation”. The ASDS has found that there is an increasing interest in the under-30 age group in preventative skincare treatments as the use of fillers and neuromodulators has surged in the past few years [2]. Future studies with larger patient populations and longer follow-up are necessary. Alternative light sources such as daylight or LED blue light may also be studied to determine protocols for maximal improvement.

## References

[1] ASDS consumer survey on cosmetic dermatologic procedures. https://www.asds.net/medical-professionals/practice-resources/asds-consumer-survey-on-cosmetic-dermatologic-procedures. Accessed 19 July 2019.

[2] ASDS members performed nearly 12 million treatments in 2017 https://www.asds.net/skin-experts/news-room/press-releases/asds-members-performed-nearly-12-million-treatments-in-2017. Accessed 19 July 2019.

[3] Almeida Issa MC, Pineiro-Maceira J, Farias RE, Pureza M, Raggio Luiz R, Manela-Azulay M (2009). Immunohistochemical expression of matrix metalloproteinases in photodamaged skin by photodynamic therapy. Br J Dermatol.

[4] Ang JM, Riaz IB, Kamal MU, Paragh G, Zeitouni NC (2017). Photodynamic therapy and pain: a systematic review. Photodiagnosis Photodyn Ther.

[5] Babes A, Sauer SK, Moparthi L (2016). Photosensitization in porphyrias and photodynamic therapy involves TRPA1 and TRPV1. J Neurosci.

[6] Bolognia J, Jorizzo JL, Schaffer JV (2012). Dermatology.

[7] Bruscino N, Lotti T, Rossi R (2011). Photodynamic therapy for a hypertrophic scarring: a promising choice. Photodermatol Photoimmunol Photomed.

[8] Fisher GJ, Kang S, Varani J (2002). Mechanisms of photoaging and chronological skin aging. Arch Dermatol.

[9] Gold MH (2002). The evolving role of aminolevulinic acid hydrochloride with photodynamic therapy in photoaging. Cutis.

[10] Goldman M, Atkin D, Kincaid S (2002). PDT/ALA in the treatment of actinic damage: real world experience. Lasers Sur Med.

[11] Issa MC, Pineiro-Maceira J, Vieira MT (2010). Photorejuvenation with topical methyl aminolevulinate and red light: a randomized, prospective, clinical, histopathologic, and morphometric study. Dermatol Surg.

[12] Ji J, Zhang LL, Ding HL (2014). Comparison of 5-aminolevulinic acid photodynamic therapy and red light for treatment of photoaging. Photodiagnosis Photodyn Ther.

[13] Le Pillouer-Prost A, Cartier H (2016). Photodynamic photorejuvenation: a review. Dermatol Surg.

[14] Lowe NJ, Lowe P (2005). Pilot study to determine the efficacy of ALA-PDT photo-rejuvenation for the treatment of facial ageing. J Cosmet Laser Ther.

[15] Lubart R, Friedmann H, Lavie R (2007). A reasonable mechanism for visible light-induced skin rejuvenation. Lasers Med Sci.

[16] Morton C, Campbell S, Gupta G (2006). Intraindividual, right-left comparison of topical methyl aminolaevulinate-photodynamic therapy and cryotherapy in subjects with actinic keratoses: a multicentre, randomized controlled study. Br J Dermatol.

[17] Ozog DM, Rkein AM, Fabi SG (2016). Photodynamic therapy: a clinical consensus guide. Dermatol Surg.

[18] Park MY, Sohn S, Lee ES, Kim YC (2010). Photorejuvenation induced by 5-aminolevulinic acid photodynamic therapy in patients with actinic keratosis: a histologic analysis. J Am Acad Dermatol.

[19] Pavicic T, Gauglitz GG, Lersch P (2011). Efficacy of cream-based novel formulations of hyaluronic acid of different molecular weights in anti-wrinkle treatment. J Drugs Dermatol.

[20] Ruiz-Rodriguez R, Lopez L, Candelas D, Pedraz J (2008). Photorejuvenation using topical 5-methyl aminolevulinate and red light. J Drugs Dermatol.

[21] Sanclemente G, Medina L, Villa JF, Barrera LM, Garcia HI (2011). A prospective split-face double-blind randomized placebo-controlled trial to assess the efficacy of methyl aminolevulinate + red-light in patients with facial photodamage. J Eur Acad Dermatol Venereol.

[22] Shin HT, Kim JH, Shim J (2015). Photodynamic therapy using a new formulation of 5-aminolevulinic acid for wrinkles in Asian skin: a randomized controlled split face study. J Dermatol Treat.

[23] Surgeons ASoP (2018) Plastic surgery statistics report. https://www.plasticsurgery.org/documents/News/Statistics/2018/plastic-surgery-statistics-full-report-2018.pdf. Accessed 01 Aug 2019.

[24] Szeimies RM, Torezan L, Niwa A (2012). Clinical, histopathological and immunohistochemical assessment of human skin field cancerization before and after photodynamic therapy. Br J Dermatol.

[25] Touma D, Yaar M, Whitehead S, Konnikov N, Gilchrest BA (2004). A trial of short incubation, broad-area photodynamic therapy for facial actinic keratoses and diffuse photodamage. Arch Dermatol.

[26] Wang B, Shi L, Zhang YF (2017). Gain with no pain? Pain management in dermatological photodynamic therapy. Br J Dermatol.

[27] Zane C, Capezzera R, Sala R, Venturini M, Calzavara-Pinton P (2007). Clinical and echographic analysis of photodynamic therapy using methylaminolevulinate as sensitizer in the treatment of photodamaged facial skin. Lasers Surg Med.

[28] Zhang HY, Ji J, Tan YM (2014). Evaluation of 5-aminolevulinic acid-mediated photorejuvenation of neck skin. Photodiagnosis Photodyn Ther.

[29] Zhang S, Duan E (2018). Fighting against skin aging: the way from bench to bedside. Cell Transpl.

